# Poor control of pain increases the risk of depression: a cross-sectional study

**DOI:** 10.3389/fpsyt.2024.1514094

**Published:** 2025-01-07

**Authors:** Junjian Zeng, Zhiqiang Liao, Aiqing Lin, Yu Zou, Yixun Chen, Zhonghua Liu, Zhidong Zhou

**Affiliations:** ^1^ Department of Anesthesiology, The Second Affiliated Hospital, Jiangxi Medical College, Nanchang University, Nanchang, Jiangxi, China; ^2^ Jiangxi Province Key Laboratory of Anesthesiology, The Second Affiliated Hospital, Jiangxi Medical College, Nanchang University, Nanchang, China

**Keywords:** depression, pain duration, pain management, pain, NHANES (National Health and Nutrition Examination Survey)

## Abstract

**Background:**

Inadequate pain management not only results in prolonged physical discomfort but also causes a range of psychological and social issues, such as anxiety, depression, social withdrawal, and diminished work performance. This study aims to investigate the relationship between the duration of pain and depression.

**Methods:**

This study utilized data on pain and depression from the National Health and Nutrition Examination Survey (NHANES) from 2011 to 2014. Participants with a health questionnaire score ≥10 were considered to have depressive symptoms. Weighted univariate, multivariate logistic regression analysis, sensitivity analysis, and restricted cubic spline (RCS) analysis were used to examine the relationship between pain duration and the risk of depression. Additionally, subgroup analysis was conducted to identify potential confounding factors that might affect this relationship.

**Results:**

Among the 2,248 participants, 442 (19.6%) were diagnosed with depressive symptoms, with an average age of 52 years, 69% of whom were female. After adjusting for all confounding factors, our results show a significant association between pain duration (in months) and depression. Individuals in the highest quartile of pain duration had a 154% higher likelihood of developing depression compared to those in the lowest quartile (OR = 3.375, 95% CI 2.329-4.886, *P* < 0.001), and the trend test was also significant (*P* for trend < 0.001). The RCS analysis indicated a linear relationship between pain duration and depression (*P* for nonlinearity = 0.427).

**Conclusion:**

This study’s results indicate that inadequate pain control, resulting in extended pain duration, places patients at a higher risk for depression.

## 1 Introduction

Depression is a prevalent and serious mental health disorder, marked by sustained feelings of sadness, loss of interest in routine activities, and various emotional and physical symptoms. According to the fifth edition of the Diagnostic and Statistical Manual of Mental Disorders (DSM-5), depression includes at least two weeks of persistent low mood and other symptoms such as fatigue, sleep disturbances, cognitive impairment, and feelings of worthlessness or guilt (1–4). The World Health Organization (WHO) estimates that more than 280 million people globally are affected by depression, positioning it as a major contributor to the global disease burden (5). Depression poses a substantial economic burden, contributing to significant healthcare expenses, productivity decline, and workday losses. On an individual level, depression exerts deep impacts on both mental and physical health (6). Individuals with depression frequently suffer from chronic fatigue, concentration difficulties, and sleep disturbances, which worsen their condition. Prior research suggests that chronic stress and prolonged inflammatory responses elevate the risk of depression by influencing multiple immune and inflammatory mechanisms (7–9).

Pain is a complex, multi-dimensional experience, encompassing both sensory and emotional aspects, usually caused by actual or potential tissue damage. The International Association for the Study of Pain (IASP) defines pain as “an unpleasant sensory and emotional experience associated with actual or potential tissue damage” (10). Pain ranks among the most frequent reasons individuals seek medical care globally, impacting about 1.5 billion people. The global cost of pain is staggering, with billions of dollars spent annually on healthcare, productivity loss, and disability compensation (11). Pain duration refers to the amount of time a person endures pain. It can range from short-term acute pain lasting a few seconds or minutes to chronic pain that endures for months or even years. Understanding pain duration is critical, as it is often associated with its effects on both mental and physical well-being. For instance, chronic pain is more frequently linked to comorbid conditions like anxiety, depression, and sleep disturbances. Moreover, the prolonged presence of pain may lead to adaptive changes in the nervous system, making treatment more difficult. There is some evidence of an association between pain and depression; however, the link between pain duration and depression needs further exploration. This cross-sectional study seeks to examine the relationship between pain duration and depression, offering insights into the etiology of depression linked to poor pain management.

## 2 Methods

### 2.1 The study population in NHANES

This study utilized data from the 2011-2014 National Health and Nutrition Examination Survey (NHANES) to evaluate the relationship between pain duration and depression in adults aged 20 and above. NHANES is a program aimed at providing a comprehensive understanding of the health and nutritional status of U.S. adults and children, employing a strict multistage probability sampling method to ensure the sample represents the entire nation’s population characteristics. All participants completed standardized household interviews and later underwent a series of assessments, including physical exams, laboratory tests, and a second interview at a Mobile Examination Center (MEC). The NHANES protocol was approved by the National Health Statistics Ethics Review Board, and all adult participants gave written informed consent. For comprehensive details on NHANES methods and ethics, please visit the CDC and NCHS websites (https://www.cdc.gov/nchs/nhanes).

**Figure 1:**
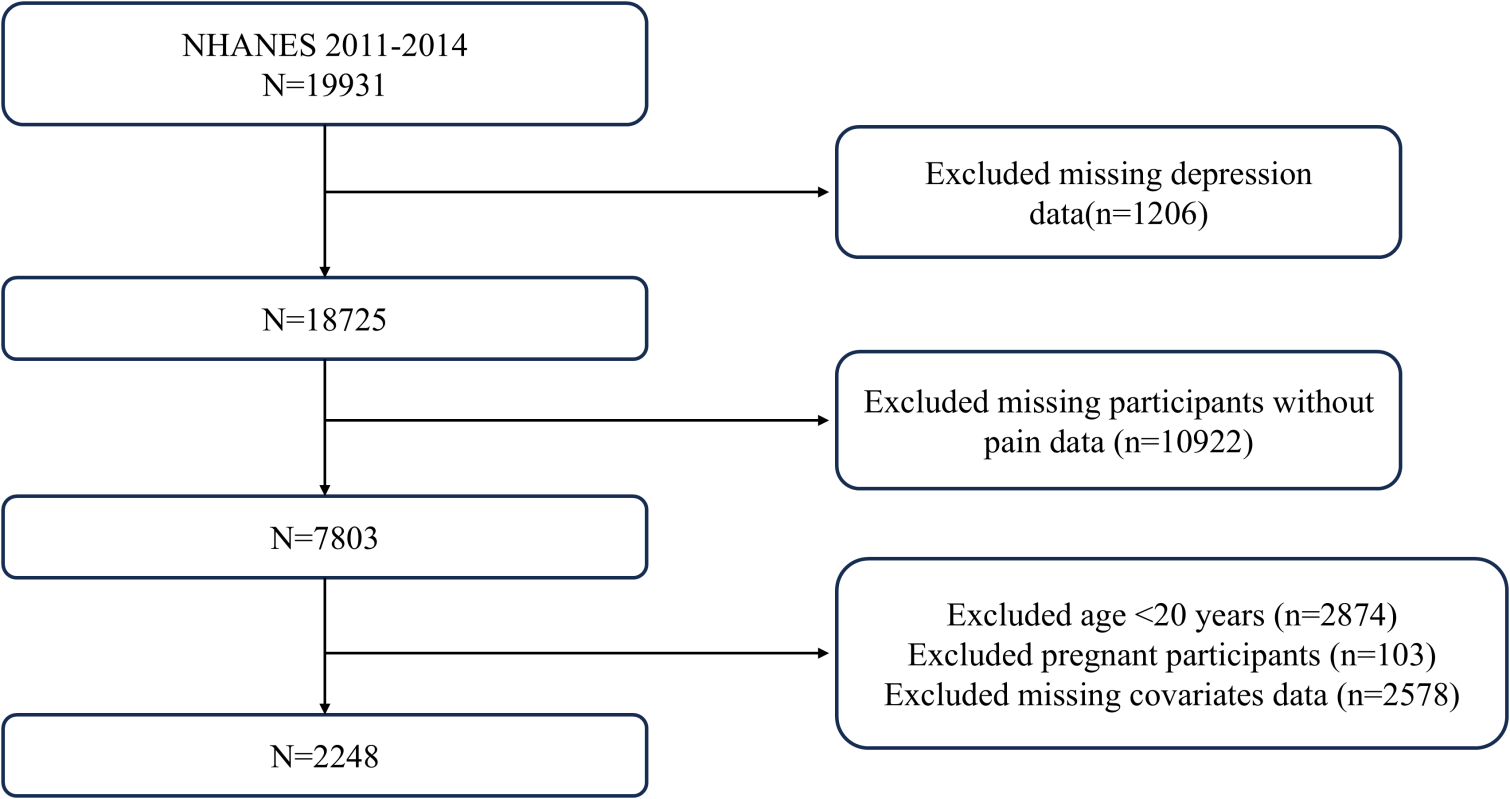
Flowchart of study selection.

In this analysis, we initially reviewed data collected from 19,931 participants during the 2011-2014 NHANES cycle. After excluding 1,206 individuals lacking depression data and 10,922 participants without pain, we narrowed the study sample to adults aged 20 and older, totaling 7,157 people. After further excluding 4,806 participants with incomplete covariate data, the final effective sample for analysis comprised 2,248 adults with pain, with 1,806 reporting no depressive symptoms and 442 reporting depressive symptoms. **Figure 1** offers a detailed visualization of the inclusion and exclusion process.

### 2.2 Assessment of depression

We employed the Patient Health Questionnaire-9 (PHQ-9) to evaluate depression. The PHQ-9 is a standardized tool designed to measure depressive symptoms experienced over the past two weeks. It includes items such as “Have little interest in doing things,” “Feeling down, depressed, or hopeless,” “Trouble sleeping or sleeping too much,” “Feeling tired or having little energy,” “Poor appetite or overeating,” “Feeling bad about yourself,” “Trouble concentrating on things,” “Moving or speaking slowly or too fast,” and “Thought you would be better off dead.” (12). This questionnaire is made up of nine questions, with each scored from 0 to 3. The sum of the scores from these nine questions results in a total score ranging from 0 to 27, where a total score of ≥10 indicates the presence of depressive symptoms (12). In this study, we defined a PHQ-9 score of less than 10 as indicating a non-depressed state, while scores of 10 or more were classified as depressive symptoms, establishing this as the criterion for the binary variable.

### 2.3 Definition of pain

To identify individuals experiencing pain and determine the duration of their pain, we used the pain questionnaire (CKQ070Q, CKQ070U), which asked participants specifically about Quantity: how long pain/aching/soreness and Unit: how long pain/aching/soreness. For responses of “I don’t know,” these were considered as no pain, while unanswered or skipped responses were treated as missing values. Based on the reported duration of pain, we converted all responses into Pain month.

### 2.4 Covariates

Based on a review of the literature and clinical practice experience, we identified the following variables as potential confounders that could influence the relationship between pain duration and depression: gender (male and female), age, race (Mexican American, non-Hispanic white, non-Hispanic Black, or other), education level (less than high school, high school or equivalent, college or higher), marital status (married/living with others, widowed/divorced/separated, never married), body mass index (BMI), ratio of family income to poverty (PIR), smoking (having smoked at least 100 cigarettes in their lifetime), alcohol use (consuming at least 12 drinks per year), hypertension (systolic blood pressure ≥ 140 mmHg and/or diastolic blood pressure ≥ 90 mmHg, self-reported hypertension), diabetes (fasting glucose ≥ 7.0 mmol/L or HbA1c ≥ 6.5%, self-reported diabetes), cardiovascular diseases (self-reported congestive heart failure, coronary heart disease, and heart attack), stroke, and cancer.

### 2.5 Statistical analysis

In this study, we accounted for the complex sampling design and weights during analysis, using the weights provided by the Mobile Examination Center (MEC) for all statistical analyses. For continuous variables with a normal distribution, we reported the mean (standard deviation, SD); for non-normally distributed variables, we provided the weighted median and interquartile range. Categorical variables were presented as unweighted frequencies and weighted percentages. As Pain month had a right-skewed distribution, we applied a natural log transformation, reporting the weighted median and interquartile range. To examine the relationship between pain duration and depression, we employed a weighted logistic regression model to estimate odds ratios (OR) and their 95% confidence intervals (CI), adjusting for potential confounders. Crude model: no covariates were adjusted. Model 1 adjusted for age, gender, race, education level, and marital status. In Model 2, we adjusted for age, gender, race, education level, marital status, PIR, BMI, smoking status, and alcohol use. Model 3, the fully adjusted model, included adjustments for all variables. By comparing models with and without interaction terms for specific confounders, we used the likelihood ratio test to evaluate the statistical significance of these interactions. Restricted cubic spline (RCS) models with four knots (5th, 35th, 65th, and 95th percentiles) were used to examine the possibility of a nonlinear relationship between pain duration and depression. Additionally, we performed subgroup analyses with stratification and interaction testing for certain covariates to assess variations across different groups. Data analysis in this study was carried out using DecisionLinnc 1.0 software (https://www.statsape.com). A two-sided *P* < 0.05 was deemed statistically significant.

## 3 Results

### 3.1 Basic characteristics of the study population

The final analysis involved 2,248 participants, with the weighted sample representing 47,373,810 people in the population. **Table 1** presents the characteristics of participants, stratified by the presence or absence of depressive symptoms. Among participants with depressive symptoms, the average age was 52 years (SD=14), 69.23% were female, and most were non-Hispanic White. In total, 442 participants (19.6%) were diagnosed with depressive symptoms. Compared to those without depressive symptoms, participants with depressive symptoms were more likely to be female, non-White, living alone or unmarried, and smokers. Furthermore, participants with depressive symptoms often had higher education levels, lower family income-to-poverty ratios, and a higher prevalence of hypertension, cardiovascular disease, and stroke (all *P* < 0.001). Due to the right-skewed distribution of Pain month data, a natural log transformation was applied, and the data was classified into quartiles. For participants with depressive symptoms, the median In-Pain month was 3.62 (**Supplementary Table S1**), suggesting that those with depression tend to experience poor pain control (*P <*0.001).

**Table 1:** Participants’ characteristics stratified by the presence of depressive symptoms.

Variables	Overall (N = 2,248)	Non-depressive symptoms (N = 1,806)	Depressive symptoms (N = 442)	*P*-value
Age, mean (SD)	53.03 (16.34)	53.04 (16.76)	52.98 (14.16)	0.939
Gender, n (%)				<0.001
Male	940 (41.73%)	804 (44.33%)	136 (29.36%)	
Female	1,308 (58.27%)	1,002 (55.67%)	306 (70.64%)	
Race, n (%)				0.013
Mexican American	25 (7.28%)	20 (7.60%)	46 (5.76%)	
Other Hispanic	20 (4.85%)	152 (4.37%)	57 (7.12%)	
Non-Hispanic White	1,020 (70.76%)	82 (71.72%)	197 (66.15%)	
Non-Hispanic Black	506 (10.46%)	402 (9.91%)	104 (13.07%)	
Other Race	26 (6.66%)	224 (6.40%)	38 (7.90%)	
Education level, n (%)				<0.001
Less than high level	53 (16.58%)	37 (14.04%)	16 (28.65%)	
High level	491 (21.62%)	396 (20.56%)	95 (26.66%)	
More than high school	1,222 (61.79%)	1,036 (65.39%)	186 (44.68%)	
Marital status, n (%)				<0.001
Married/living with partner	359 (14.74%)	293 (14.83%)	66 (14.30%)	
Widowed/divorced/separated	1,244 (62.42%)	1,059 (65.01%)	185 (50.13%)	
Never married	645 (22.83%)	454 (20.16%)	191 (35.56%)	
Body mass index, mean (SD)	30.17 (7.50)	29.69 (7.22)	32.15 (8.52)	<0.001
PIR, mean (SD)	2.27 (1.62)	2.43 (1.62)	1.61 (1.37)	<0.001
Hypertension, n (%)	814 (32.30%)	643 (31.91%)	171 (34.19%)	0.248
Hyperlipidemia, n (%)	1,117 (51.49%)	888 (51.82%)	229 (49.93%)	0.346
Cardiovascular disease, n (%)	232 (8.52%)	149 (7.08%)	83 (15.35%)	<0.001
Diabetes, n (%)	336 (10.52%)	248 (9.58%)	88 (15.00%)	0.001
Smoking, n (%)	1,136 (52.12%)	858 (48.88%)	278 (67.50%)	<0.001
Drinking, n (%)	767 (38.45%)	631 (39.86%)	136 (31.73%)	0.109
Stroke, n (%)	114 (4.09%)	72 (3.37%)	42 (7.53%)	<0.001
Cancer, n (%)	292 (15.27%)	225 (15.03%)	67 (16.40%)	0.151
Pain month, median (IQR)	11.37(0.23-60.83)	6.20(0.23-48.67)	36.50(6.20-133.83)	<0.001
In Pain month, median (IQR)	2.52(0.21-4.12)	1.97(0.21-3.91)	3.62(1.97-4.90)	<0.001
In Pain month, n (%)				<0.001
Quartile 1	969 (42.50%)	863 (46.72%)	106 (22.42%)	
Quartile 2	507 (21.95%)	408 (21.83%)	99 (22.52%)	
Quartile 3	518 (23.06%)	363 (21.02%)	155 (32.78%)	
Quartile 4	254 (12.49%)	172 (10.43%)	82 (22.28%)	

All analyses have been weighted to account for the survey's complex sampling design.

### 3.2 Correlation between pain duration and depression

**Figure 2:**
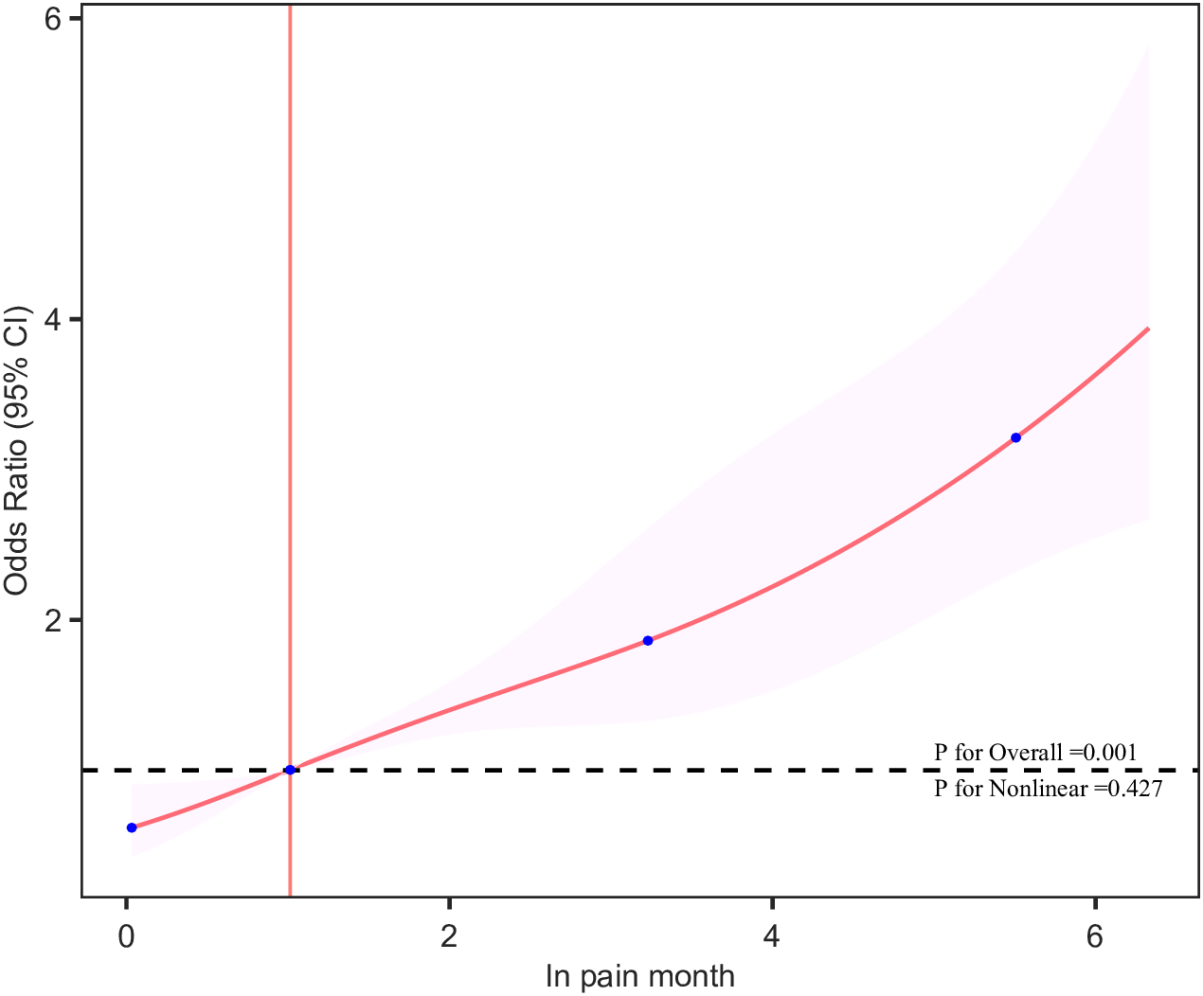
The dose–response relationships of In Pain month with the prevalence of depression. The solid red line represents the smooth curve fit between variables. The shaded bands represent the 95% confidence intervals. The intersection of the pink line is the cutoff point.

**Table 2** presents the results of univariate logistic regression analysis, showing the associations between age, race, education level, PIR, smoking, hypertension, diabetes, Pain month, In-Pain month, and depression. Using In Pain month as the independent variable and depression incidence as the dependent variable, a multivariate logistic regression analysis was conducted to evaluate the association between In-Pain month and the incidence of depression (**Table 3**). The results indicated that the continuous variable In-Pain month was significantly associated with the incidence of depression. This correlation was observed in both the crude model (OR = 1.335, 95% CI 1.263-1.412, *P* < 0.001) and the fully adjusted model (OR = 1.295, 95% CI 1.217-1.378, *P* < 0.001). Specifically, each unit increase in In-Pain month was associated with a 29% increase in depression incidence. To further analyze, we transformed In-Pain month from a continuous variable to a categorical variable (Quartiles), using Quartile 1 as the reference, and calculated the OR and 95% CI for Quartiles 2, 3, and 4 using logistic regression. In the crude model, the depression incidence in Quartile 4 was significantly higher than in Quartile 1 (OR = 3.881, 95% CI 2.783-5.405, *P* < 0.001). In the fully adjusted model, this positive correlation remained robust (OR = 3.375, 95% CI 2.329-4.886, *P* < 0.001). Furthermore, *P* for trend < 0.001, which further confirms the statistically significant trend that with increasing pain duration, the risk of depression also increases, suggesting this is not a random occurrence. In all three models, compared to Quartile 1, Quartiles 2 and 3 also showed significant changes in depression incidence (*P* < 0.001), suggesting a linear relationship between In Pain month and depression. The multivariate-adjusted RCS model demonstrated a linear dose-response relationship between pain duration and the risk of depressive symptoms (*P* = 0.001, *P* for nonlinear = 0.0427, **Figure 2**). These results suggest that poor pain control leads to prolonged pain duration, significantly increasing the risk of depression among participants.

**Table 2:** Bivariate associations between variables and depressive symptoms.

Variables	OR (95% CI)	*P*-value
Age	1.000(0.994-1.006)	0.945
Gender		
Male	1(Ref)	
Female	1.805(1.448-2.260)	<0.001
Race		
Mexican American	1(Ref)	
Other Hispanic	1.671(1.076-2.607)	0.023
Non-Hispanic White	1.067(0.753-1.536)	0.722
Non-Hispanic Black	1.153(0.788-1.707)	0.470
Other Race	0.756(0.471-1.208)	0.243
Education_level		
Less than high level	1(Ref)	
High level	0.557(0.416-0.744)	<0.001
More than high school	0.417(0.327-0.531)	<0.001
Marital status		
Married/living with partner	1(Ref)	
Widowed/divorced/separated	0.776(0.572-1.062)	0.107
Never married	1.868(1.368-2.575)	<0.001
Body mass index	1.039(1.026-1.052)	<0.001
PIR	0.667(0.613-0.724)	<0.001
Hypertension	1.141(0.920-1.413)	0.227
Hyperlipidemia	1.111(0.903-1.369)	0.320
Cardiovascular_disease	2.571(1.915-3.434)	<0.001
Diabetes	1.562(1.189-2.037)	0.001
Smoking	1.873(1.514-2.323)	<0.001
Drinking	0.828(0.660-1.033)	0.098
Stroke	2.529(1.691-3.739)	<0.001
Cancer	1.255(0.929-1.678)	0.131
Pain month	1.003(1.002-1.004)	<0.001
In pain month	1.335(1.264-1.412)	<0.001
In pain month		
Quartile 1	1(Ref)	
Quartile 2	1.976(1.465-2.662)	<0.001
Quartile 3	3.476(2.642-4.589)	<0.001
Quartile 4	3.881(2.784-5.406)	<0.001

Results are based on weighted data. Abbreviations: OR: odds ratio; CI: confidence interval; Ref: reference.

**Table 3:** Association between In Pain month and depression in multiple regression model.

Variables (N=2248)	Crude Model		Model 1		Model 2		Model 3	
	OR(95% CI)	*P*-value	OR(95% CI)	*P*-value	OR(95% CI)	*P*-value	OR(95% CI)	*P*-value
In pain month	1.335(1.263-1.412)	<0.001	1.362(1.284-1.446)	<0.001	1.296(1.219-1.379)	<0.001	1.295(1.217-1.378)	<0.001
Categorical variable								
Quartile 1	1(Ref)		1(Ref)		1(Ref)		1(Ref)	
Quartile 2	1.975(1.465-2.662)		1.957(1.439-2.660)	<0.001	1.800(1.310-2.473)	<0.001	1.835(1.332-2.527)	<0.001
Quartile 3	3.476(2.642-4.589)		3.455(2.601-4.604)	<0.001	2.912(2.172-3.917)	<0.001	2.915(2.168-3.931)	<0.001
Quartile 4	3.881(2.783-5.405)		4.433(3.125-6.289)	<0.001	3.406(2.365-4.901)	<0.001	3.375(2.329-4.886)	<0.001
*P* for trend	<0.001		<0.001		<0.001		<0.001	

Results are based on weighted data. Abbreviations: OR: odds ratio; CI: confidence interval; Ref: reference.

Crude model: No covariates are adjusted.

Model 1: Adjusted for age, sex, race, education level, and marital status.

Model 2: Adjusted for age, sex, race, education level, marital status, ration of family income to poverty, body mass index, smoking, and drinking.

Model 3: Adjusted for age, sex, race, education level, marital status, ration of family income to poverty, body mass index, smoking, drinking, hypertension, hyperlipidemia, diabetes, cardiovascular disease, stroke and cancer.

### 3.3 Stratified analysis

Stratified analyses were conducted to assess the influence of various factors on the association between pain duration and depression, taking into account age, BMI, PIR, and others. The results from these groups indicated no significant interactions (**Supplementary Figure S1**).

### 3.4 Sensitivity analysis

In the sensitivity analysis, we excluded 254 participants with extreme pain duration, resulting in a remaining cohort of 1994 individuals. In the fully adjusted model, when compared to Quartile 1, the adjusted odds ratios (OR) for In-Pain month and depression in Quartiles 2, 3, and 4 were 1.867 (95% CI 1.246-2.824, *P* < 0.001), 2.515 (95% CI 1.702-3.762, *P* < 0.001), and 3.919 (95% CI 2.660-5.853, *P* < 0.001), respectively (**Supplementary Table S2**). These results indicate that the relationship between pain duration and depression remained stable even after excluding extreme values. In particular, the association between pain duration and depression remained consistent, with inadequate pain control elevating the risk of depression.

## 4 Discussion

This research involves a cross-sectional analysis of the depressed population within the NHANES database. The objective is to investigate the association between pain duration and depression. The findings reveal a notable positive correlation between pain duration and depression. Even after adjusting for various confounding factors, this correlation remains significant, with dose-response analysis further demonstrating a linear relationship between pain duration and depression. Previous studies on pain and depression have primarily focused on the relationship between the presence or absence of pain and depression, or on the relationship between chronic pain and depression. Most of these studies concentrate on the existence or non-existence of pain, with limited attention to the specific effects of pain duration. Our research addresses this gap, emphasizing the importance of promptly relieving and eliminating pain in individuals experiencing pain to prevent the negative consequences of chronic pain.

While the causes of depression are complex, recent research has highlighted multiple contributing factors. These factors encompass genetic predispositions, biochemical imbalances, psychosocial stressors, and environmental effects. Advances in neuroscience have identified that alterations in neurotransmitter systems, especially serotonin, norepinephrine, and dopamine, are crucial in the development of depression (13, 14). The mechanism suggests that chronic stress can initiate inflammatory responses, where pro-inflammatory cytokines like interferon-γ and tumor necrosis factor-α affect the synthesis of crucial monoamines such as serotonin and dopamine, thereby influencing the onset and progression of depression (15), emphasizing the role of stress-related biological processes. Recent research has highlighted the importance of inflammatory and immune indicators derived from peripheral blood counts, such as the neutrophil-lymphocyte ratio (NLR), monocyte-lymphocyte ratio (MLR), and platelet-lymphocyte ratio (PLR) as biomarkers for depression (16, 17). These indicators not only assist in the diagnosis of depression but also offer crucial insights into disease progression and treatment responses.

Pain may be an important stressor that leads to depression. Acute pain typically occurs alongside an inflammatory response that activates pain receptors, which are specifically designed to detect harmful stimuli and transmit pain signals to the brain’s nerve endings. Conversely, chronic pain encompasses more intricate mechanisms, such as the sensitization of the nervous system, where the body becomes increasingly sensitive to pain signals. This means that pain can act as both an acute and chronic stressor that triggers inflammatory responses. Inflammatory conditions are closely linked to the development of mood disorders, including depression, complicating the challenges of pain management (18, 19). Pain can greatly impact a patient’s emotional well-being, while depression may worsen symptoms in those with pain and diminish treatment effectiveness. A high comorbidity rate exists between the two pathologies, which may share certain common pathological mechanisms. Zhu et al. found that individuals with comorbid pain and depression show notable differences in socioeconomic status, lifestyle, and behavioral patterns compared to those with only one of the conditions (20). A study of American adults revealed that 23.9% of chronic pain patients experience unresolved anxiety or depression symptoms, compared to only 4.9% in those without chronic pain (21). Likewise, data from the elderly population show a comparable trend, with approximately 28.4% of older adults with chronic pain exhibiting depressive symptoms (22, 23). These findings highlight the crucial need for timely alleviation or eradication of pain among patients suffering from it. Poor pain management can lead to prolonged pain duration, which in turn can trigger anxiety or depressive symptoms, sleep disturbances, cognitive decline, and impaired social relationships, further exacerbating patient suffering. Our study is the first to explore the relationship between the long-term impact of pain and depression, rather than being limited to chronic pain, providing a new perspective on understanding pain-related mental disorders. Similarly, our results suggest that there are deficiencies in current pain management practices. Many patients fail to achieve effective pain relief, potentially due to existing treatment modalities such as medication, physical therapy, and psychological interventions, which, though effective, may not be applicable to all patients. Additionally, many patients lack knowledge about pain management and self-care, hindering their ability to actively engage in their treatment (24). Effective pain management not only alleviates patient suffering but also enhances their quality of life and functional status. Effective pain management can aid in minimizing the incidence of complications, including cardiovascular diseases and impaired immune function (25–27). Thus, a holistic approach to pain management, integrating pharmacological treatment, non-pharmacological interventions, and psychological support, is essential for enhancing the overall health of patients. Future studies should investigate more effective pain management strategies and develop tailored treatment options to better tackle this intricate health challenge.

However, the current study has some limitations. First, as NHANES is a cross-sectional study, it cannot establish causal relationships or long-term impacts; future studies should use a prospective longitudinal design to better confirm the link between the long-term effects of pain and depression. Second, the diagnosis of depression and pain data were obtained through self-reported questionnaires, which may be influenced by recall bias; future studies should integrate various assessment tools and expert interviews with clinicians to enhance data accuracy and reliability. Lastly, because there is a lack of information regarding the use of antidepressants and pain medications in data from U.S. adults between 2011 and 2014, future research should further investigate the impact of these drugs on pain patients with coexisting depression. This will contribute to a more thorough understanding of how pharmacological treatments play a role in this intricate relationship.

## 5 Conclusion

Our study results show that In Pain month is significantly associated with depression linearly, regardless of potential confounding factors. This insight suggests that poor pain management results in prolonged pain duration, increasing the risk of depression for patients. Future studies should focus on clarifying the specific mechanisms that connect the long-term effects of pain with depression.

## Data Availability

The original contributions presented in the study are included in the article/**Supplementary Material**. Further inquiries can be directed to the corresponding author.
