# Supplementary material for: Poor control of pain increases the risk of depression: a cross-sectional study
Source: Front Psychiatry. 2025 Jan 7;15:1514094. doi: 10.3389/fpsyt.2024.1514094 (PMC11747233; doi:10.3389/fpsyt.2024.1514094)
Supplement: Supplementary file 1 [file SupplementaryFile1.docx]

|  | | | | |
| --- | --- | --- | --- | --- |
| **Variables** | **Overall (N = 2,248)** | **Non-depressive symptoms(N = 1,806)** | **Depressive symptoms(N = 442)** | ***P*-value** |
|  |  |  |  |  |
| Age, mean (SD) | 53.03 (16.69) | 53.04 (17.25) | 52.98 (14.22) | 0.939 |
| Gender, n (%) |  |  |  | <0.001 |
| Male | 940 (41.81%) | 804 (44.52%) | 136 (30.77%) |  |
| Female | 1,308 (58.19%) | 1,002 (55.48%) | 306 (69.23%) |  |
| Race, n (%) |  |  |  | 0.013 |
| Mexican American | 25 (11.17%) | 20 (11.35%) | 46 (10.41%) |  |
| Other Hispanic | 20 (9.30%) | 152 (8.42%) | 57 (12.90%) |  |
| Non-Hispanic White | 1,020 (45.37%) | 82 (45.57%) | 197 (44.57%) |  |
| Non-Hispanic Black | 506 (22.51%) | 402 (22.26%) | 104 (23.53%) |  |
| Other Race | 26 (11.65%) | 224 (12.40%) | 38 (8.60%) |  |
| Education level, n (%) |  |  |  | <0.001 |
| Less than high level | 53 (23.80%) | 37 (20.71%) | 16 (36.43%) |  |
| High level | 491 (21.84%) | 396 (21.93%) | 95 (21.49%) |  |
| More than high school | 1,222 (54.36%) | 1,036 (57.36%) | 186 (42.08%) |  |
| Marital status, n (%) |  |  |  | <0.001 |
| Married/living with partner | 359 (15.97%) | 293 (16.22%) | 66 (14.93%) |  |
| Widowed/divorced/separated | 1,244 (55.34%) | 1,059 (58.64%) | 185 (41.86%) |  |
| Never married | 645 (28.69%) | 454 (25.14%) | 191 (43.21%) |  |
| Body mass index, mean (SD) | 30.17 (7.70) | 29.69 (7.30) | 32.15 (8.89) | <0.001 |
| PIR, mean (SD) | 2.27 (1.57) | 2.43 (1.61) | 1.61 (1.21) | <0.001 |
| Hypertension, n (%) | 814 (36.21%) | 643 (35.60%) | 171 (38.69%) | 0.248 |
| Hyperlipidemia, n (%) | 1,117 (49.69%) | 888 (49.17%) | 229 (51.81%) | 0.346 |
| Cardiovascular disease, n (%) | 232 (10.32%) | 149 (8.25%) | 83 (18.78%) | <0.001 |
| Diabetes, n (%) | 336 (14.95%) | 248 (13.73%) | 88 (19.91%) | 0.001 |
| Smoking, n (%) | 1,136 (50.53%) | 858 (47.51%) | 278 (62.90%) | <0.001 |
| Drinking, n (%) | 767 (34.12%) | 631 (34.94%) | 136 (30.77%) | 0.109 |
| Stroke, n (%) | 114 (5.07%) | 72 (3.99%) | 42 (9.50%) | <0.001 |
| Cancer, n (%) | 292 (12.99%) | 225 (12.46%) | 67 (15.16%) | 0.151 |
| Pain month, median (IQR) | 12.17(0.23-60.83) | 6.20 (0.23 - 48.67) | 36.50 (5.17 - 121.67) | <0.001 |
| In Pain month, median (IQR) | 2.58(0.21-4.12) | 1.97(0.21-3.91) | 3.62(1.82-4.81) | <0.001 |
| In Pain month, n (%) |  |  |  | <0.001 |
| Quartile 1 | 969 (43.10%) | 863 (47.79%) | 106 (23.98%) |  |
| Quartile 2 | 507 (22.55%) | 408 (22.59%) | 99 (22.40%) |  |
| Quartile 3 | 518 (23.04%) | 363 (20.10%) | 155 (35.07%) |  |
| Quartile 4 | 254 (11.30%) | 172 (9.52%) | 82 (18.55%) |  |
|  | | | | |

**Table S1. Unweighted population characteristics by the presence of depressive symptoms.**

|  |  |  | |  | | | | |  |
| --- | --- | --- | --- | --- | --- | --- | --- | --- | --- |
| **Variables**  **(N=1994)** | **Crude Model** | | **Model 1** | | | **Model 2** | | **Model 3** | |
|  | **OR(95% CI)** | ***P*-value** | **OR(95% CI)** | | ***P*-value** | **OR(95% CI)** | ***P*-value** | **OR(95% CI)** | ***P*-value** |
| In pain month | 1.392(1.298-1.494) | <0.001 | 1.390(1.293-1.497) | | <0.001 | 1.296(1.219-1.379) | <0.001 | 1.328(1.232-1.434) | <0.001 |
| Categorical variable |  |  |  | |  |  |  |  |  |
| Quartile 1 | 1(Ref) |  | 1(Ref) | |  | 1(Ref) |  | 1(Ref) |  |
| Quartile 2 | 2.135(1.461-3.154) |  | 2.137(1.450-3.184) | | <0.001 | 2.005(1.343-3.021) | <0.001 | 1.867(1.246-2.824) | <0.001 |
| Quartile 3 | 2.890(2.007-4.218) |  | 2.877(1.976-4.245) | | <0.001 | 2.588(1.754-3.865) | <0.001 | 2.515(1.702-3.762) | <0.001 |
| Quartile 4 | 5.179(3.615-7.536) |  | 5.124(3.532-7.545) | | <0.001 | 4.068(2.766-6.064) | <0.001 | 3.919(2.660-5.853) | <0.001 |
| *P* for trend | <0.001 |  | <0.001 | |  | <0.001 |  | <0.001 |  |
|  |  |  | |  | | | | |  |

**Table S2. The relationship between In Pain month and depression in individuals who do not have excessively prolonged pain.**

Note: Results are based on weighted data. Abbreviations: OR: odds ratio; CI: confidence interval; Ref: reference.

Crude model: No covariates are adjusted.

Model 1: Adjusted for age, sex, race, education level, and marital status.

Model 2: Adjusted for age, sex, race, education level, marital status, ration of family income to poverty, body mass index, smoking, and drinking.

Model 3: Adjusted for age, sex, race, education level, marital status, ration of family income to poverty, body mass index, smoking, drinking, hypertension, hyperlipidemia, diabetes, cardiovascular disease, stroke and cancer.


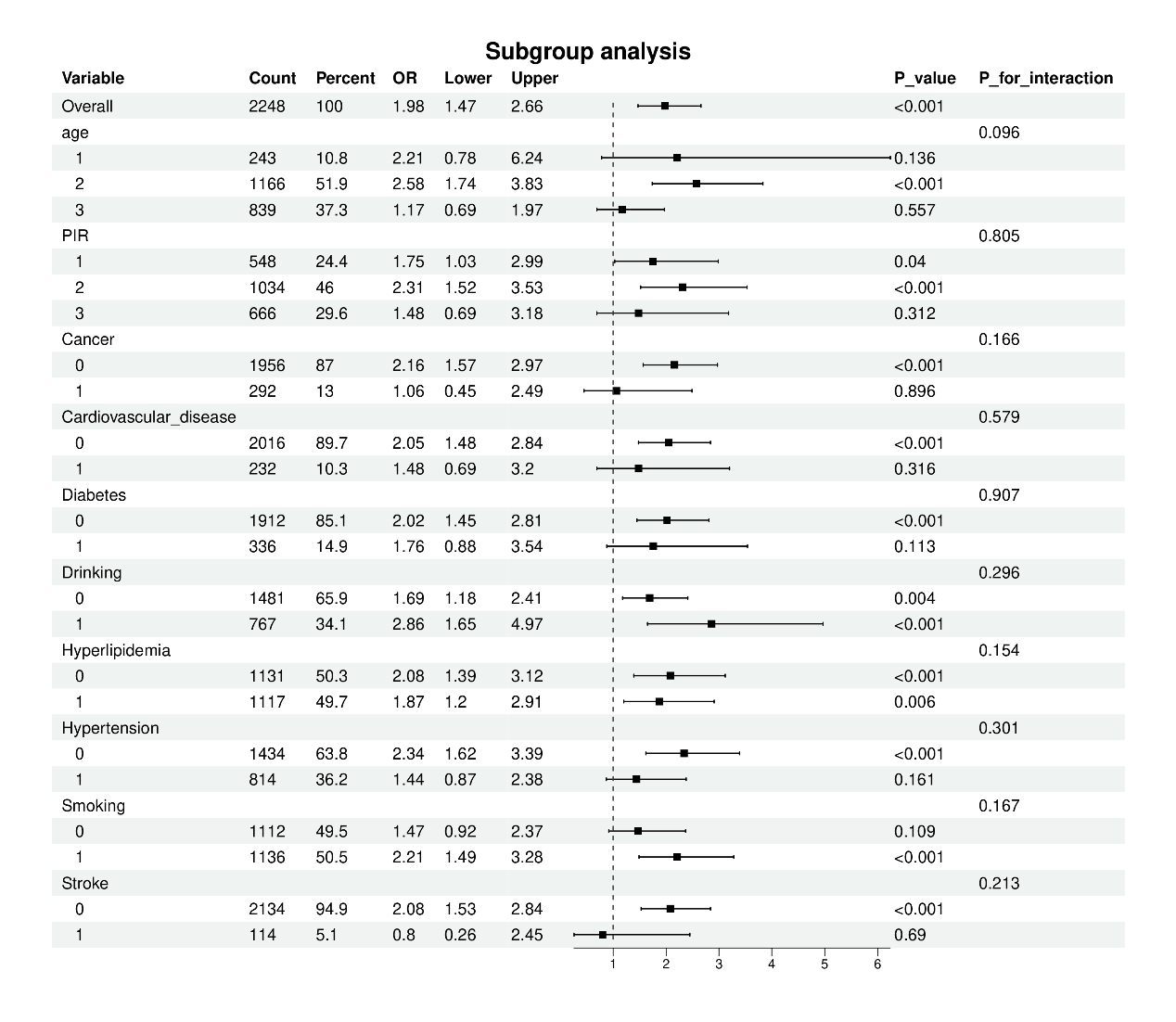


**Figure S1. Subgroup analysis of the interaction between In Pain month and depressive symptoms.**

Note: *P* for interaction represents the likelihood of interaction between the variables and depressive symptoms.

Abbreviations: OR: odds ratio; CI: confidence interval; Ref: reference.
